# Use of biologicals in allergic and type-2 inflammatory diseases during the current COVID-19 pandemic: Position paper of Ärzteverband Deutscher Allergologen (AeDA)^A^, Deutsche Gesellschaft für Allergologie und Klinische Immunologie (DGAKI)^B^, Gesellschaft für Pädiatrische Allergologie und Umweltmedizin (GPA)^C^, Österreichische Gesellschaft für Allergologie und Immunologie (ÖGAI)^D^, Luxemburgische Gesellschaft für Allergologie und Immunologie (LGAI)^E^, Österreichische Gesellschaft für Pneumologie (ÖGP)^F^ in co-operation with the German, Austrian, and Swiss ARIA groups^G^, and the European Academy of Allergy and Clinical Immunology (EAACI)^H^

**DOI:** 10.5414/ALX02166E

**Published:** 2020-09-07

**Authors:** Ludger Klimek, Oliver Pfaar, Margitta Worm, Thomas Eiwegger, Jan Hagemann, Markus Ollert, Eva Untersmayr, Karin Hoffmann-Sommergruber, Alessandra Vultaggio, Ioana Agache, Sevim Bavbek, Apostolos Bossios, Ingrid Casper, Susan Chan, Alexia Chatzipetrou, Christian Vogelberg, Davide Firinu, Paula Kauppi, Antonios Kolios, Akash Kothari, Andrea Matucci, Oscar Palomares, Zsolt Szépfalusi, Wolfgang Pohl, Wolfram Hötzenecker, Alexander R. Rosenkranz, Karl-Christian Bergmann, Thomas Bieber, Roland Buhl, Jeroen Buters, Ulf Darsow, Thomas Keil, Jörg Kleine-Tebbe, Susanne Lau, Marcus Maurer, Hans Merk, Ralph Mösges, Joachim Saloga, Petra Staubach, Uta Jappe, Klaus F. Rabe, Uta Rabe, Claus Vogelmeier, Tilo Biedermann, Kirsten Jung, Wolfgang Schlenter, Johannes Ring, Adam Chaker, Wolfgang Wehrmann, Sven Becker, Laura Freudelsperger, Norbert Mülleneisen, Katja Nemat, Wolfgang Czech, Holger Wrede, Randolf Brehler, Thomas Fuchs, Peter-Valentin Tomazic, Werner Aberer, Antje-Henriette Fink-Wagner, Fritz Horak, Stefan Wöhrl, Verena Niederberger-Leppin, Isabella Pali-Schöll, Wolfgang Pohl, Regina Roller-Wirnsberger, Otto Spranger, Rudolf Valenta, Mübecell Akdis, Paolo M. Matricardi, François Spertini, Nicolai Khaltaev, Jean-Pierre Michel, Larent Nicod, Peter Schmid-Grendelmeier, Marco Idzko, Eckard Hamelmann, Thilo Jakob, Thomas Werfel, Martin Wagenmann, Christian Taube, Erika Jensen-Jarolim, Stephanie Korn, Francois Hentges, Jürgen Schwarze, Liam O´Mahony, Edward F. Knol, Stefano del Giacco, Tomás Chivato Pérez, Jean Bousquet, Anna Bedbrook, Torsten Zuberbier, Cezmi Akdis, Marek Jutel

**Affiliations:** 1 *Zentrum für Rhinologie und Allergologie, Wiesbaden, *; 2 *HNO-Universitätsklinik Marburg, Sektion Rhinologie und Allergologie, Medizinische Fakultät Marburg, Philipps-Universität Marburg, *; 3 *Comprehensive Allergy Centre Charité, Klinik für Dermatologie, Venerologie und Allergologie, Charité – Universitätsmedizin Berlin, Germany, *; 4 *Translational Medicine Program, Peter Gilgan Centre for Research and Learning, Hospital for Sick Children, Toronto, Ontario, Canada, *; 5 *Division of Immunology and Allergy, Food Allergy and Anaphylaxis Program, The Hospital for Sick Children, Toronto, Ontario, Canada, *; 6 *Department of Immunology, University of Toronto, Toronto, Ontario, Canada, *; 7 *Hals-, Nasen-, Ohrenklinik und Poliklinik, Universitätsmedizin Mainz, Germany *; 8 *Department of Infection and Immunity, Luxembourg Institute of Health (LIH), Esch-sur-Alzette, Luxemburg, *; 9 *Department of Dermatology and Allergy Center, Odense Research Center for Anaphylaxis, University of Southern Denmark, Odense, Denmark, *; 10 *Institute of Pathophysiology and Allergy Research, Center of Pathophysiology, Infectiology and Immunology, Medizinische Fakultät der Universität Wien, Vienna, Austria, *; 11 *Immunoallergology Unit, Careggi University Hospital, Florence, Italy, *; 12 *Transylvania University, Brasov, Romania, *; 13 *Ankara University, School of Medicine, Department of Chest Disease, Division of, Immunology and Allergy, Ankara, Turkey, *; 14 *Abteilung für Atemwegsmedizin und Allergie, Karolinska University Hospital, Huddinge und Abteilung für Medizin, Huddinge, Karolinska Institutet, Stockholm, Sweden, *; 15 *Zentrum für Allergieforschung, Karolinska Institutet, Stockholm, Sweden, *; 16 *Department of Immunology, University Hospital Zürich, Zürich, Switzerland, *; 17 *Faculty of Medicine, University of Zürich, Zürich, Switzerland, *; 18 *Guy’s and St. Thomas’ NHS Foundation Trust, Westminster Bridge Road, London, United Kingdom, King’s College London School of Life Course Sciences & School of Immunology & Microbial Sciences, King’s Health Partners, United Kingdom, *; 19 *Allergy Unit 2*; 20 * Department of Dermatology and Venereology, National University of Athens, Medical School, University General Hospital „ATTIKON“, Athen, Greece, *; 21 *Universitätsklinikum Carl Gustav Carus, Klinik und Poliklinik für Kinder- und Jugendmedizin, Fachbereich Kinderpneumologie/Allergologie, Dresden, Germany *; 22 *Department of Medical Sciences and Public Health, University of Cagliari, Monserrato, Italy, *; 23 *Abteilung für Allergie, Entzündungszentrum, Universitätsklinikum Helsinki, Helsinki, Finland, *; 24 *Department of Medicine, Beth Israel Deaconess Medical Center, Harvard Medical School, Boston, Massachusetts, USA, *; 25 *Department of Biochemistry and Molecular Biology, Chemistry School, Complutense University of Madrid, Spain, *; 26 *Abteilung für Pädiatrische Pulmologie, Allergologie und Endokrinologie, Universitätsklinik für Kinder- und Jugendheilkunde, Comprehensive Center for Pediatrics, Medizinische Universität Wien, Vienna, Austria, *; 27 *Abteilung für Atmungs- und Lungenerkrankungen, Krankenhaus Hietzing, Vienna, Austria, *; 28 *Abteilung für Dermatologie und Venerologie, Kepler Universitätsklinikum, Linz, Austria, *; 29 *Klinische Abteilung für Nephrologie, Universitätsklinik für Innere Medizin, Medizinische Universität Graz, Graz, Austria, *; 30 *Klinik für Dermatologie und Allergologie, Universität Bonn, Bonn, *; 31 *Schwerpunkt Pneumologie, III. Medizinische Klinik und Poliklinik, Universitätsmedizin Mainz, Mainz, *; 32 *Zentrum Allergie und Umwelt (ZAUM) Technische Universität und Helmholtz Zentrum München, *; 33 *Klinik und Poliklinik für Dermatologie und Allergologie der Technischen Universität München, *; 34 *Institut für Klinische Epidemiologie und Biometrie, Universität Würzburg, *; 35 *Allergie- und Asthma-Zentrum Westend, Berlin, *; 36 *Klinik für Pädiatrie m.S. Pneumologie, Immunologie und Intensivmedizin, Charité – Universitätsmedizin Berlin, *; 37 *Abteilung Dermatologie und Allergologie, RWTH Universität, Aachen, *; 38 *Medizinische Fakultät der Universität zu Köln, Cologne *; 39 *CRI – Clinical Research International Ltd., Hamburg, *; 40 *ClinCompetence Cologne GmbH, Köln, Cologne *; 41 *Hautklinik und Poliklinik, Universitätsmedizin Mainz, *; 42 *Forschungsgruppe Klinische und Molekulare Allergologie des Forschungszentrums Borstel, Airway Research Center North (ARCN), Mitglied des Deutschen Zentrums für Lungenforschung (DZL); Interdisziplinäre Allergie-Ambulanz, Medizinische Klinik III, Universität zu Lübeck, *; 43 *LungenClinic Grosshansdorf, Großhansdorf, *; 44 *Klinik für Allergologie, Johanniter-Krankenhaus im Fläming Treuenbrietzen GmbH, Treuenbrietzen, *; 45 *Klinik für Innere Medizin Schwerpunkt Pneumologie, Philipps-Universität Marburg, *; 46 *Einheit für Klinische Allergologie (EKA), Helmholtz Zentrum München, German Research Center for Environmental Health GmbH, Neuherberg, *; 47 *Praxis für Dermatologie, Immunologie und Allergologie, Erfurt, *; 48 *Ärzteverband Deutscher Allergologen, Dreieich, *; 49 *Haut- und Laserzentrum an der Oper, München, Munich, *; 50 *Academia München, *; 51 *HNO-Klinik, Universitätsklinik TUM, München, *; 52 *ZAUM, Helmholtz Zentrum München, Munich *; 53 *Praxis für Dermatologie und Allergologie, Münster, *; 54 *Klinik für Hals-, Nasen- und Ohrenheilkunde, Universität Tübingen, *; 55 *Asthma und Allergiezentrum Leverkusen, *; 56 *Klinik für Kinder- und Jugendmedizin, Universitätsklinikum Carl Gustav Carus, Dresden; Praxis für Kinderpenumologie/Allergologie am Kinderzentrum Dresden (Kid), Dresden, *; 57 *Praxis und Klinik für Dermatologie/Allergologie am Schwarzwald-Baar Klinikum, Villingen-Schwenningen, *; 58 *Hals-, Nasen- und Ohrenarzt, Nordrhein-Westfalen, *; 59 *Universitätsklinikum Münster, Klinik für Hautkrankheiten, Ambulanz für Allergologie, Berufsdermatologie und Umweltmedizin, Münster, *; 60 *Klinik für Dermatologie, Venerologie und Allergologie, Universitätsklinikum, Georg-August-Universität, Göttingen, Germany *; 61 *Klinische Abteilung für allgemeine HNO, Medizinische Universität Graz, Austria, *; 62 *Universitätsklinik für Dermatologie und Venerologie, Medizinische Universität Graz, Austria, *; 63 *Global Allergy and Airways Patient Platform GAAPP, Vienna, Austria, *; 64 *Allergiezentrum Wien West, Vienna, Austria, *; 65 *Floridsdorfer Allergiezentrum, Vienna, Austria, *; 66 *Universitätsklinik für Hals-, Nasen- und Ohrenkrankheiten, Medizinische Universität Vienna, Austria, *; 67 *Komperative Medizin, Interdisziplinäres Messerli Forschungsinstitut, Veterinärmedizinische Universität Wien, Medizinische Universität Wien, *; 68 *Institut für Pathophysiologie und Allergieforschung, Medizinische Universität Wien, *; 69 *Abteilung für Atmungs- und Lungenerkrankungen, Krankenhaus Hietzing, Vienna, Austria, *; 70 *Universitätsklinik für Innere Medizin, Medizinische Universität Graz, Austria, *; 71 *Österreichische Lungenunion, Vienna, Austria, *; 72 *Immunopathologie, Abteilung für Pathophysiologie und Allergieforschung, Zentrum für Pathophysiologie, Infektiologie und Immunologie, Medizinische Universität Wien, Austria, *; 73 *Swiss Institute of Allergy and Asthma Research (SIAF), University of Zurich, Davos, Switzerland, *; 74 *Charité – Universitätsmedizin Berlin, *; 75 *Division of Allergy and Immunology, Centre Hospitalier Universitaire Vaudois, Lausanne, Switzerland, *; 76 *GARD Chairman, Genf, Switzerland, *; 77 *Department of Rehabilitation and Geriatrics, University of Geneva, Genf, Switzerland, *; 78 *Clinic Cecil of Hirslanden Group of Lausanne; Centre Hôpitalier Universitaire du canton de Vaud Lausanne, Switzerland, *; 79 *Allergiestation, Dermatologische Klinik, Universitätsspital Zürich, Switzerland, *; 80 *Klinische Abteilung für Pneumologie, Universitätsklinik für Innere Medizin II, Medizinische Universität Wien, Austria, *; 81 *Kinderzentrum Bethel, Evangelisches Klinikum Bethel, Universitätsmedizin OWL der Universität Bielefeld, *; 82 *Klinik für Dermatologie und Allergologie, Universitätsklinikum Gießen, UKGM, Justus-Liebig-Universität, Gießen, *; 83 *Klinik für Dermatologie, Allergologie und Venerologie Medizinische Hochschule Hannover, *; 84 *HNO-Klinik, Universitätsklinikum Düsseldorf, *; 85 *Universitätsklinikum Essen (AöR), Germany *; 86 *Service Immunologie-Allergologie Centre Hospitalier de Luxembourg, Luxemburg, *; 87 *Kinderleben und Gesundheit, Universität von Edinburgh, United Kingdom, *; 88 *Medicine and Microbiology, APC Microbiome Ireland, National University of Ireland, Cork, Irland, *; 89 *Departments of Immunology, Dermatology and Allergology, University Medical Center Utrecht, the Netherlands, *; 90 *Università degli Studi di Cagliari, Cagliari, Italy, *; 91 *University Foundation San Pablo CEU, Madrid, Spain, *; 92 *MACVIA-France, Fondation partenariale FMC VIA-LR, Montpellier, France, *; 93 *INSERM U 1168, VIMA: Ageing and chronic diseases Epidemiological and public health approaches, Villejuif, France, *; 94 *Université Versailles St-Quentin-en-Yvelines, UMR-S 1168, Montigny le Bretonneux, France, *; 95 *Euforea, Brussels, Belgium, *; 96 *Berlin Institute of Health, Comprehensive Allergy Center, Department of Dermatology and Allergy, Charité, Universitätsmedizin Berlin, Humboldt-Universität zu Berlin, Germany *; 97 *Department of Clinical Immunology, Wrocław Medical University, Wrocław, Poland, *; 98 *ALL-MED Medical Research Institute, Wrocław, Poland, and *; 99 *Dermatologische Allergologie, Allergie-Centrum-Charité, Klinik für Dermatologie, Venerologie und Allergologie, Charité – Universitätsmedizin Berlin, Germany*; A *Ärzteverband Deutscher Allergologen (AeDA)*; B *Deutsche Gesellschaft für Allergologie und Klinische Immunologie (DGAKI)*; C *Gesellschaft für Pädiatrische Allergologie und Umweltmedizin (GPA)*; D *Österreichische Gesellschaft für Allergologie und Immunologie (ÖGAI)*; E *Luxemburgische Gesellschaft für Allergologie und Immunologie (LGAI)*; F *Österreichische Gesellschaft für Pneumologie (ÖGP)*; G *German, Austrian, and Swiss ARIA groups*; H *European Academy of Allergy and Clinical Immunology (EAACI)*; *Authors are co-first authors.; **Authors are co-last authors.

**Keywords:** COVID-19, SARS-CoV-2, telemedicine, dupilumab, omalizumab, benralizumab, reslizumab, mepolizumab

## Abstract

Background: Since the beginning of the COVID-19 pandemic, the treatment of patients with allergic and atopy-associated diseases has faced major challenges. Recommendations for “social distancing” and the fear of patients becoming infected during a visit to a medical facility have led to a drastic decrease in personal doctor-patient contacts. This affects both acute care and treatment of the chronically ill. The immune response after SARS-CoV-2 infection is so far only insufficiently understood and could be altered in a favorable or unfavorable way by therapy with monoclonal antibodies. There is currently no evidence for an increased risk of a severe COVID-19 course in allergic patients. Many patients are under ongoing therapy with biologicals that inhibit type 2 immune responses via various mechanisms. There is uncertainty about possible immunological interactions and potential risks of these biologicals in the case of an infection with SARS-CoV-2. Materials and methods: A selective literature search was carried out in PubMed, Livivo, and the internet to cover the past 10 years (May 2010 – April 2020). Additionally, the current German-language publications were analyzed. Based on these data, the present position paper provides recommendations for the biological treatment of patients with allergic and atopy-associated diseases during the COVID-19 pandemic. Results: In order to maintain in-office consultation services, a safe treatment environment must be created that is adapted to the pandemic situation. To date, there is a lack of reliable study data on the care for patients with complex respiratory, atopic, and allergic diseases in times of an imminent infection risk from SARS-CoV-2. Type-2-dominant immune reactions, as they are frequently seen in allergic patients, could influence various phases of COVID-19, e.g., by slowing down the immune reactions. Theoretically, this could have an unfavorable effect in the early phase of a SARS-Cov-2 infection, but also a positive effect during a cytokine storm in the later phase of severe courses. However, since there is currently no evidence for this, all data from patients treated with a biological directed against type 2 immune reactions who develop COVID-19 should be collected in registries, and their disease courses documented in order to be able to provide experience-based instructions in the future. Conclusion: The use of biologicals for the treatment of bronchial asthma, atopic dermatitis, chronic rhinosinusitis with nasal polyps, and spontaneous urticaria should be continued as usual in patients without suspected infection or proven SARS-CoV-2 infection. If available, it is recommended to prefer a formulation for self-application and to offer telemedical monitoring. Treatment should aim at the best possible control of difficult-to-control allergic and atopic diseases using adequate rescue and add-on therapy and should avoid the need for systemic glucocorticosteroids. If SARS-CoV-2 infection is proven or reasonably suspected, the therapy should be determined by weighing the benefits and risks individually for the patient in question, and the patient should be involved in the decision-making. It should be kept in mind that the potential effects of biologicals on the immune response in COVID-19 are currently not known. Telemedical offers are particularly desirable for the acute consultation needs of suitable patients.

Revised and updated version of the following EAACI position paper, including an adaption to the situations in Germany, Austria, Switzerland, and Luxembourg: Vultaggio A et al.: Considerations on Biologicals for Patients with allergic disease in times of the COVID-19 pandemic: an EAACI Statement. Allergy. 2020 (in press).

**German version published in Allergologie, Vol. 43, No. 7/2020, pp. 255-271** [Table Abbreviation]

## Introduction 

The clinical symptoms of infection with the novel coronavirus (severe acute respiratory coronavirus 2; SARS-CoV-2) became known as the “coronavirus disease 2019 (COVID-19)” on February 11, 2020 [1]. The International Committee on Taxonomy of Viruses (ICTV) called this novel human pathogenic virus SARS-CoV-2 [1]. The global spread of the SARS-CoV-2 pandemic and patients with severe COVID-19 courses pose a major challenge to healthcare systems worldwide. 

The coronavirus that caused the severe acute respiratory syndrome (SARS-CoV) in 2002/2003 has approximately an 80% nucleotide sequence identity with SARS-CoV-2 [1]. SARS-CoV-2 is a betacoronavirus of the subgenus Sarbecovirus, subfamily Orthocoronavirinae, and the 7^th^ member of the Coronaviridae family that can infect humans. It can be isolated from human samples obtained from respiratory secretions, nasal and pharyngeal swabs and isolated on cell cultures [1, 2, 3]. 

It is covered by a lipid membrane that can be disrupted by detergents and is different from the Middle East respiratory syndrome-related coronavirus (MERS-CoV), from SARS-CoV, and from the coronaviruses responsible for the common cold (229E, OC43, NL63, and HKU1) [1]. 

The incubation period after an infection with SARS-CoV-2 can be of up to 14 days, during which the infected person can be asymptomatic but nevertheless transmit the virus. 

In a high number of patients, the infection leads to symptoms of the upper and lower airways, and, less frequently, also of other organ systems (nervous system, gastrointestinal tract, kidneys, blood vessels). In the worst case scenario, multi-organ failure and respiratory failure can result, as has been described for other coronavirus infections (SARS-CoV-1, MERS-CoV) [4, 5, 6]. In more severe cases, infection with SARS-CoV-2 can lead to pneumonia, severe acute respiratory syndrome, renal failure, and death [4, 7, 8, 9, 10]. Higher age and comorbidities such as chronic airway diseases (particularly COPD), diabetes mellitus, cardiovascular diseases, obesity, and immune deficiency of various origins have been described as risk factors of a severe course [4, 7, 8, 9]. The need for intensive care treatment and invasive ventilation is associated with high mortality. 

We will present clinical and immunological aspects that have to be considered with regard to the COVID-19 pandemic in patients treated with biological therapy against IgE and mediators of type 2 inflammation (Table 1). 

## Immune response in SARS-CoV-2 and other coronavirus infections 

The characteristics of the immune response after infection with SARS-CoV-2 are still insufficiently understood. While various forms of the course of COVID-19 and the infection with the virus have been described, it is still unclear which immunological background influences the course of the disease. This is also true for the role of the innate and adaptive immune system with regard to SARS-CoV-2 infection. While natural killer (NK) cells traditionally play an important role in the early phase of viral infections, CD8^+^ T helper cells come into action in the subsequent phase [11]. Early antibody secretion and production in the mucosa-associated lymphatic tissue initially include antigen-specific IgM, IgA, and, later, IgG antibodies, and are essential for immune response [12, 13, 14]. Macrophages are activated and secrete inflammatory cytokines, with type 1 interferons (type 1 IFN) playing the most prominent role. In infections with other coronaviruses (e.g., SARS-CoV-1), type 1 IFN is responsible for the adequate initiation of the immune reaction, and patients with delayed or insufficient IFN production have a more severe disease course [6]. 

The activation of apoptosis or pyroptosis in epithelial cells serves as an antiviral defense, but excessive immune reactions can also contribute to local tissue damage through synergistic effects [15]. An excessive production of pro-inflammatory cytokines has already been observed in SARS-CoV-1, MERS-CoV, and recently also in SARS-CoV-2 infections, and has been described as a cytokine storm [4, 5]. Natural IgM, and probably also mannose-binding lectin (MBL), are believed to be the first line of defense against SARS-CoV-2 [16]. These antibodies and MBL recognize glycans and are abundant in children and young adults. However, they decrease dramatically with age and are over 50 times lower at the age of ≥ 60 than at the age of 20 – 30 years [16]. As they are part of the innate immunity, they are the only antibodies able to recognize SARS-CoV-2 before the adaptive immune response is initiated [16]. If the virus enters the lungs early enough, it can replicate in an unhindered manner, as no or only little resistance exists. The resulting inflammation with a massive activation of local mediators (complement and coagulation cascades interleukin-6 (IL-6), cytokine storm) can cause damages that lead to complications or, in some cases, even to death [16]. 

Furthermore, the ability of SARS-CoV-2 viruses to penetrate the cell via receptors such as ACE2 and TMPRRSS2 could explain the different severities of COVID-19 in different patient groups [17]. 

**Figure 1.**
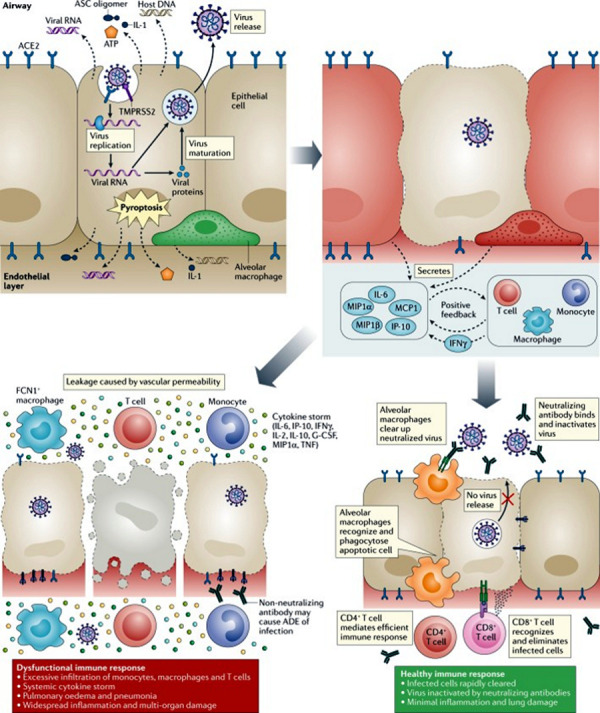
Sequence of immunological events in SARS-CoV-2 infection: If SARS-CoV-2 infects cells via the surface receptors ACE2 and TMPRSS2, this leads to active replication and release of the virus, the cells decay through pyroptosis. DAMPs are released that are recognized by neighboring epithelia, endothelia, and alveolar macrophages and trigger the release of pro-inflammatory cytokines such as IL-6, IP-10, MIP1α, MIP1β, and MCP1. This attracts monocytes, macrophages, and T cells, which, when IFN-γ is added, activate another inflammatory, self-reinforcing cascade. In defective immune responses (left), this can lead to accumulation of immune cells and overproduction of pro-inflammatory cytokines, which then damage the lung and may lead to a cytokine storm with multi-organ failure. Additionally, non-neutralizing antibodies produced by B cells may enhance the infection and lead to further organ damage. In a healthy immune response (right), the initial inflammation attracts virus-specific T cells to the infection site where they can eliminate the infected cells before the virus spreads further. Neutralizing antibodies selectively block the virus, alveolar macrophages recognize and phagocytize affected cells and neutralized viruses. Altogether, these processes clear the viral infection with minimal damage of respiratory tissue and lead to recovery. Reproduced from Tay et al. [65]. ACE2 = angiotensin-converting enzyme 2; ADE = antibody-dependent enhancement; DAMP = damage-associated molecular pattern; G-CSF = granulocyte colony-stimulating factor; IFN = interferon; IL = interleukin; MIP1α = macrophage inflammatory protein 1α; SARS-CoV-2 = severe acute respiratory syndrome coronavirus 2; TNF = tumor necrosis factor.

Extensive damage to the lungs leads to rapid clinical deterioration and usually to the need for intensive care treatment, which can be observed typically 7 –14 days after infection. The risk of kidney, liver, and/or other organ damage as well as of consumption coagulopathy is significantly increased. Affected patients usually have highly increased interleukin (IL)-1 β, IL-6, IL-8, and TNF-α levels (Figure 1) [18]. The therapeutic blockade of one or more of these cytokines has been discussed as a potential future therapy option for severely affected patients in whom IL-6 can be massively increased [19]. IL-6 plays a central role in the cytokine storm, and tocilizumab has already been used as a biological with anti-IL-6 effects in COVID-19 [20, 21]. 

Approved indications for anti-IL-6 or anti-IL-6R antibodies (such as tocilizumab, sarilumab) currently include, for example, rheumatoid arthritis, juvenile rheumatoid arthritis, and Castleman disease. The immune reactions of type 1 and type 3 described here are contained by other cytokines, such as IL-10 and TGF- β, and type 2 inflammation could possibly counteract the cytokine storm. Increased levels of eosinophilic granulocytes, as one of the key cells of type 2 inflammation, have been ascribed a protective effect in severe viral infections, although the mechanism of action has not yet been identified [22]. On the other hand, low blood eosinophil counts could simply reflect the severity of the infection. The interaction of SARS-CoV-2 with its receptor on the cells of the respiratory system, the membrane-bound angiotensin-converting enzyme 2 (ACE2), which is responsible for the entry of the virus into the host cell, is far better investigated [17]. 

Therefore, the reduced expression of ACE2 in the airway epithelial cells of patients with allergic asthma is being discussed as a potentially protective factor against SARS-CoV-2 infection [23]. It can be assumed that only the interaction of the individual cytokine responses leads to an adequate and effective immune response in coronavirus infections. However, imbalances between type 1, type 2, and type 3 reactions might have a significant negative or positive impact on the course of the viral infection (Figure 1). 

## Experiences with COVID-19 in diseases involving type 2 inflammation 

So far, there is insufficient evidence to indicate which risk factors cause a severe course of COVID-19. A history of lung disease has been considered a potential risk factor for developing COVID-19 and possibly also for a severe course. Bronchial asthma, which is the most important allergic indication for biologicals targeting IgE and type 2 inflammation, is possibly one of these diseases. However, since many patients with pulmonary disease also have other comorbidities, some of the suspected factors could turn out to be confounders once further studies are carried out. Thus, it remains unclear whether patients with type-2-associated bronchial asthma without any other possible risk factors should be considered high risk for a severe COVID-19 course. The currently available data rather contradict this. There is only limited data on COVID-19 in connection with a type-2-associated disease, and its prognostic value is very limited. The currently available studies do not indicate an increased risk for patients with allergies, asthma, or other atopy-associated diseases (Table 2) [8, 24, 25, 26, 27, 28, 29]. For example, in Wuhan and Italy, the percentage of seriously ill or deceased COVID-19 patients with known bronchial asthma was far below the prevalence of asthma in these places [18]. It also remains unclear why in many patients not only lymphopenia but also eosinopenia was detected at the time of admission [8]. Neither decreased nor increased eosinophil levels have so far been clearly associated with certain clinical courses of SARS-CoV-2 infection. 

## Marketing approval of biologicals in type 2 inflammation in Germany, Austria, Luxembourg, and Switzerland 

In recent years, several biologicals have been approved in Europe that block IgE antibodies or the interleukins IL-4, IL-5, and IL-13, which are relevant in type 2 inflammation, or their receptors [30, 31, 32, 33, 34, 35, 36, 37, 38, 39]. 

Omalizumab has been approved for the treatment of severe allergic bronchial asthma in adults and in children older than 6 years with proven sensitization against a perennial airborne allergen and reduced lung function. Another indication is antihistamine-resistant chronic spontaneous urticaria in adults and in adolescents older than 12 years. Mepolizumab, benralizumab, and reslizumab are IL-5 or IL-5 receptor blockers, available for adults and, partially, also for children. 

Dupilumab has been approved for the treatment of: (i) atopic dermatitis in adolescents older than 12 years, (ii) severe type-2-dominant asthma in adults and (iii) chronic rhinosinusitis with nasal polyps (CRSwNP) in adults. 

Table 2 shows the situation of approval in Germany, Austria, Luxembourg, and Switzerland. Self-administration by the patient is listed separately as it significantly facilitates care for suitable patients during the SARS-CoV-2 pandemic. 

## Type 2 inflammation blocking and viral infections 

Viral infections of the upper and lower airways have been associated with the development and exacerbation of allergic disease [40, 41, 42]. Infection and the persistence of virus particles in the mucosa could inhibit the efficacy of the local innate immune system and promote type 2 inflammation. The blocking of type 2 inflammation by therapeutic antibodies against IgE, IL-5, or the IL-5 or IL-4/-13 receptors has so far not been suspected to increase the risk of viral infections. However, IL-4 has a dual role in viral infections due to two different haplotypes in the IL-4 gene. It can promote infections with the Herpes virus and the norovirus [43] as well as with the Ebola virus, which is related to the coronavirus [44]. On the other hand, IL-4 can also inhibit viral infections by promoting innate immunity [45, 46, 47]. [Table Table3]

Thus, more evidence from clinical observations is necessary to be able to provide clear recommendations with regard to COVID-19. Table 4 gives an overview of the frequency of viral infections occurring as adverse events in trials on these monoclonal antibodies. There have been reports on the lower incidence of viral infections under anti-IgE treatment with omalizumab, since this therapy may increase the functionality and the production of IFN-α by plasmacytoid dendritic cells (pDC). This leads to an enhanced antiviral defense and to a reduction of virus-induced asthma exacerbations [40, 48]. Also, for type 2 blockade with anti-IL-5 antibodies (mepolizumab, reslizumab) or anti-IL-5 receptor antibodies (benralizumab), the risk of respiratory viral infections in the active-agent study groups was equal to or lower than the risk in the placebo groups (Table 4). 

## Type 2 inflammation blocking in SARS-CoV-2 infections 

It has not yet been clarified whether the blockade of type 2 inflammation or of IgE influences the risk of developing COVID-19 or its course. In the case of a cytokine storm, possible negative effects induced by blocking the type 2 immune response situation are conceivable; but these effects require further investigation. The first reports show that the disease course is not worse in COVID-19 patients with eosinophilic diseases under biological therapy [24, 49]. However, further study results should be awaited, especially considering the fact that SARS-CoV-2 changes rapidly due to mutations [50]. 

Meta-analyses by Agache et al. [51, 52, 53] have shown a slightly increased rate (low to medium risk of association) of adverse events when anti-IL-5/5R, anti-IL-4/13R, and anti-IgE are used in severe asthma, independently of COVID-19. Thus, there is no clear recommendation regarding the decision-making to continue or temporarily interrupt a biological therapy during an infection with SARS-CoV-2. Treatment interruption could entail the risk of suboptimal control of the allergic disease or, in the case of exacerbations, the need for systemic glucocorticosteroids, for which an increased risk of a possibly more severe COVID-19 course has been described [54]. 

## Recommendations for the management of allergic/atopy-associated diseases under anti-type-2 therapy during the COVID-19 pandemic (Table 1) 

To ensure an appropriate, high-quality, and accurate care for patients on anti-type-2 treatment with underlying atopic-eosinophilic or allergic disease, antibody therapy should be continued and remain unchanged during the ongoing pandemic when there is no evidence of SARS-CoV-2 infection. To cope with the current shortfalls in hospitals and the more difficult hygiene conditions, telephone or telemedical follow-up should be considered in suitable patients when technical and medical requirements allow for it. For this purpose, comprehensive patient training with regard to documentation of the disease activity and, where applicable, to self-administration of the medication is desirable. This is facilitated by the partial availability of user-friendly pen systems for self-application. 

In general, in countries with low infection numbers and a consequent relaxation of COVID-19-associated restrictions, there is no contraindication for starting biological therapy in patients without evidence of a current SARS-CoV-2 infection. 

According to the current state of knowledge, biological therapy for the indications discussed here can be continued in mild to moderate cases of SARS-CoV2 infection/COVID-19 disease, if an individual consideration of risks and benefits supports this decision. 

The risks and benefits have to be assessed by a specialist, and it is recommended to inform the patient about the fact that only limited data are available. 

In severe courses of COVID-19, prolongation of the dosing interval or treatment interruption should be considered. When doing so, the risk of the potential requirement of treatment with systemic glucocorticoids should also be taken into account. In a quarantine situation, a telemedical approach might be feasible, in particular with the aim of continuing or expanding the basic therapy with topical steroids, inhaled bronchodilators, antihistamines, etc. in accordance with the relevant guideline recommendations [36, 37, 54, 55, 56, 57, 58]. 

If hospitalization due to the exacerbation of asthma- or type-2-associated diseases becomes necessary, current guidelines on diagnosis and treatment must be followed. Sinus surgery for CRSwNP should, if possible, be delayed in patients with suspected or confirmed COVID-19 disease. 

In the case of urgently indicated systemic therapy for severe atopic dermatitis, consideration should be given to therapy with either biologicals, classic immunosuppressants, or systemic glucocorticosteroids, although systemic glucocorticosteroids are not recommended due to their broad immunosuppressive effect (see above). For cyclosporin A (CyA) as an approved therapeutic option for atopic dermatitis, in vitro studies have suggested antiviral effects [60]. T-cell-directed immunosuppression performed after organ transplantation (CyA, tacrolimus) is being discussed as a possible protective factor against serious clinical complications of SAS-CoV-2 infection [61], as well as the use of CyA in COVID-19 [62, 63]. However, reliable clinical data have not yet been published. Possible metabolic interactions between CyA and lopinavir/ritonavir (CYP3 inhibitors) have to be taken into account. Severe COVID-19 courses have been reported in two patients with atopic dermatitis treated with dupilumab [64]. 

## Conclusion 

The currently available data suggest that the risk of developing a severe course of COVID-19 is probably not increased in patients with allergies and atopy-associated diseases. However, there is a lack of study results including subgroup analyses on seriously ill atopy patients and their treatment. The effects of IgE or type 2 inflammation blocking on SARS-CoV-2 infection have not yet been clarified. 

In cases of a mild to moderate COVID-19 course, we advise to continue biological therapy for the indications mentioned here, if the patient-based assessment of the benefits and risks supports this approach and if the patient agrees after adequate information about the limited availability of data. 

In severe courses of COVID-19, prolongation of the dosing interval or treatment interruption should be considered for the indications discussed here. This assessment should be patient-based and should consider the risk of the possible requirement of systemic glucocorticosteroids. 

In all other patients, in whom neither a suspected nor a proven SARS-CoV-2 infection is present, the use of biologicals for the treatment of bronchial asthma, atopic dermatitis, CRSwNP, and spontaneous urticaria can be continued unchanged or can be re-started in the current SARS-CoV-2 pandemic. 

The use of telemedicine for treatment support and patient education is recommended and can facilitate the continuation of biological administration by self-injection. 

## Conflict of interest 

E. Untersmayr, I. Agache, S. Bavbek, I. Casper, S. Chan, A. Chatzipetrou, W. Pohl, T. Bieber, T. Keil, J. Kleine-Tebbe, J. Saloga, P. Staubach, U. Rabe, C. Vogelmeier, K. Jung, J. Ring, W. Wehrmann, S. Becker, L. Freudelsperger, K. Nemat, H. Wrede, T. Fuchs, V. Niederberger-Leppin, W. Pohl, R. Roller-Wirnsberger, P.M. Matricardi, F. Spertini, N. Khaltaev, L. Nicod, M. Idzko, E. Hamelmann, T. Jakob, C. Taube, L. O´Mahony, S. del Giacco, T. Zuberbier, C. Akdis, M. Jutel, T. Eiwegger, K.-C. Bergmann, M. Akdis, O. Spranger, N. Mülleneisen, A.-H. Fink-Wagner, K.F. Rabe, W. Czech, S. Wöhrl, J. Buters, F. Horak, W. Schlenter, I. Pali-Schöll, A. Matucci, A. Vultaggio, Z. Szepfalusi, C. Vogelberg, T. Werfel, U. Jappe, J.-P. Michel, P. Kauppi, A. Chaker, E.F. Knol, T. Chivato Pérez, K. Hoffmann-Sommergruber, A.R. Rosenkranz, W. Hötzenecker, M. Ollert, A. Kothari, W. Aberer, A. Kolios, D. Firinu, P.-V. Tomazic, E. Jensen-Jarolim, F. Hentges, and A. Bedbrook state that no conflicts of interest exist. 

R. Mösges received personal fees and/or grants from Allergopharma, Allergy Therapeutics, Bencard, Leti, Lofarma, Stallergenes, Optima, Friulchem, Hexal, Klosterfrau, FAES, Meda, Novartis, UCB, BitopAG, Hulka, Ursapharm, Menarini, Mundipharma, Pohl-Boskamp, Inmunotek, Hikma, Sandoz, Lek, Cassella, SanofiGenzyme, Engelhard, SmartPeakFlow, and Strathos outside the submitted work. 

M. Maurer received grants/research support and/or personal, consultancy, or speaker fees from Amgen, Allakos, Celldex, CSL Behring, FAES, Genentech, Lilly, Merckle Recordati, Moxie, Novartis, Roche, Sanofi, Takeda, MSD, UCB, and Uriach outside the submitted work. 

P. Schmid-Grendelmeier received speaker and personal fees from AstraZeneca, GSK, Novartis Pharma, and Sanofi-Genzyme outside the submitted work. 

M. Worm received personal and/or consultancy fees from ALK-Abelló Arzneimittel GmbH, Mylan Deutschland GmbH, Leo Pharma GmbH, Sanofi-Aventis Deutschland GmbH, Regeneron Pharmaceuticals, DBV Technologies SA, Stallergenes GmbH, HAL Allergie GmbH, Allergopharma GmbH & Co.KG, Bencard Allergie GmbH, Aimmune Therapeutics UK Limited, Actelion Pharmaceuticals Deutschland GmbH, Novartis AG, Biotest AG, AbbVie Deutschland GmbH & Co. KG, and Lilly Deutschland GmbH outside the submitted work. 

O. Pfaar received personal fees and/or grants from ALK-Abelló, Allergopharma, Stallergenes Greer, HAL Allergy Holding BV/HAL Allergie GmbH, Bencard Allergie GmbH/Allergietherapeutika, Lofarma, Biomay, Circassia, ASIT Biotech Tools SA, Laboratorios LETI/LETI Pharma, MEDA Pharma/MYLAN, Anergis SA, Mobile Chamber Experts (einem GA2LEN-Partner), Indoor Biotechnologies, Glaxo Smith Kline, Astellas Pharma Global, EUFOREA, ROXALL, NOVARTIS, SANOFI AVENTIS, Med Update Europe GmbH, streamedup! outside the submitted work. 

H. Merk reports grants and/or personal fees from Meda, Stallergenes, Sanofi, Bayer, BMS, J & J outside the submitted work. 

S. Lau is a member of the Advisory Board of Sanofi Aventis. 

S. Korn received speaker and personal adviser’s fees from AstraZeneca, GSK, Novartis, and Sanofi outside the submitted work. 

U. Darsow was a lecturer, principal investigator, and consultant for ALK Abello, Bencard, and Novartis Pharma outside the submitted work. 

O. Palomares received research grants and/or personal fees from Allergy Therapeutics, Amgen, AstraZeneca, Diater, GlaxoSmithKline, S.A., Inmunotek S.L., Novartis, Sanofi-Genzyme, and Stallergenes outside the submitted work; he was a member of advisory boards of Novartis and Sanofi-Genzyme. 

T. Biedermann was a consultant to or received personal lecture fees or research grants from Alk-Abelló, Celgene-BMS, Lilly Deutschland GmbH, Mylan, Novartis, Phadia-Thermo Fisher, Sanofi-Genzyme, Regeneron. 

R. Valenta received research grants from Viravaxx, Vienna, Austria, and from HVD Life Sciences, Vienna, Austria, and acts as a consultant for Viravaxx. 

R. Buhl received personal lecture and/or consultant fees from AstraZeneca, Boehringer Ingelheim, Chiesi, Cipla, Novartis, Roche, Sanofi, and Teva as well as research support for Universitätsmedizin Mainz from Boehringer Ingelheim, GlaxoSmithKline, Novartis, and Roche, outside the submitted work. 

R. Brehler received personal fees for lectures and/or consultancy and/or clinical studies from ALK, Allergopharma, Almirall, Astra Zeneca, Bencard, Gesellschaft zur Förderung der Dermatologischen Forschung und Fortbildung e.V., Gesellschaft für Information und Organisation mbH, GSK, Dr. Pfleger, HAL, Leti, Merck, Novartis, Oto-Rhino-Laryngologischer Verein, Pierre Fabre, Pohl Boskamp, Stallergenes, Thermo-Fischer, Biotech Tools, Genentech, Circassia. 

A. Bossios reports personal consultant and/or lecture fees from Novartis, AstraZeneca, GSK, and TEVA outside the submitted work. 

J. Bousquet reports personal fees from Chiesi, Cipla, Hikma, Menarini, Mundipharma, Mylan, Novartis, Sanofi-Aventis, Takeda, Teva, Uriach, KYomed-Innov, and Purina outside the submitted work. 

J. Schwarze received personal fees from MYLAN, F2F events; support from industry for educational acivities of the Scottish Allergy and Respiratory Academy as well as the Children and Young People’s Allergy Network Scotland outside the submitted work; support from industry for EAACI; J. Schwarze is EAACI Secretary General 2019 – 2021. 

J. Hagemann received speaker fees from Novartis Pharma. 

M. Wagenmann received personal consultant and/or speaker fees from ALK-Abelló, Allergopharma, AstraZeneca, Bencard-Allergie, Genzyme, HAL-Allergie, Infectopharm, LETI Pharma, MEDA Pharma, Novartis, Sanofi Aventis, Stallergenes, Teva. 

L. Klimek reports grants and/or personal fees from Allergopharma, MEDA/Mylan, HAL Allergie, ALK Abelló, LETI Pharma, Stallergenes, Quintiles, Sanofi, ASIT Biotech, Lofarma, Allergy Therapeut., AstraZeneca, GSK, Inmunotk outside the submitted work; he is a member of the following organizations: AeDA, DGHNO, Deutsche Akademie für Allergologie und klinische Immunologie, HNO-BV GPA, EAACI. 

**Abbreviations Abbreviation:** Abbreviations

ACE2	Angiotensin-converting enzyme 2
COVID-19	Coronavirus disease 2019
CRSwNP	Chronic rhinosinusitis with nasal polyps
CyA	Cyclosporin A
ICTV	International Committee on Taxonomy of Viruses
IL	Interleukin
MBL	Mannose-binding lectin
MERS	Middle East respiratory syndrome
NK cells	Natural killer cells
pDC	Plasmacytoid dendritic cells
SARS	Severe acute respiratory syndrome
SARS-CoV	Severe acute respiratory syndrome coronavirus
SARS-CoV-2	Severe acute respiratory syndrome coronavirus 2
Type 1 IFN	Type 1 interferon

**Table 1.** Recommendations on treatment with biologicals during the COVID-19 pandemic in patients with asthma, atopic dermatitis, urticaria, or CRSwNP.

Recommendations for biological treatment in non-infected patients during the COVID-19 pandemic or in patients who have recovered from COVID-19 infection	Recommendations for biological treatment in patients with diagnosed or suspected SARS-CoV-2 infection
Termination of biological treatment is not generally necessary; and biologicals should be continued as scheduled, particularly in severe cases, based on an individual risk-benefit analysis.	In mild-to-moderate COVID-19 courses, or when SARS-CoV-2 infection is suspected, biologicals can be continued in the indications discussed here if a patient-based risk-benefit analysis supports the decision and the patient consents after having been informed about the limited availability of data.
Prolongation of the injection interval can be considered (as indicated in the summary of product characteristics) to limit the necessary physician-patient contacts to a minimum.	In severe COVID-19 courses, prolongation of the injection interval (as indicated in the summary of product characteristics) or treatment interruption should be considered in the indications discussed here. The risk of the possible requirement of systemic glucocorticosteroids must be considered.
Biological treatment can be continued during the current COVID-19 pandemic in asymptomatic patients with negative PCR tests, in patients without known exposure or contact with SARS-CoV-2-positive people, and in patients who have completed an adequate quarantine period.	In a quarantine situation, telemedical support might be feasible, in particular with the aim of continuing the basic therapy with topical steroids, inhaled bronchodilators, antihistamines, etc. in accordance with the relevant guideline recommendations or with the aim to expand those therapies according to the patient’s needs.
Biological therapy in patients without evidence of SARS-CoV-2 infection can be started for approved indications	
During the current COVID-19 pandemic, self-administration of biologicals should generally be preferred; this is made easier if user-friendly pen systems for self-application are available. Adequate patient training is required.	
Practices and allergy centers must be prepared for the current COVID-19 pandemic by following the recommendations of the WHO and of national and regional authorities.	
These recommendations should be continuously updated and adapted to new scientific findings and recommendations made by authorities.	

CRSwNP = chronic rhinosinusitis with nasal polyps.

**Table 2.** Studies including patients with COVID-19 and a history of allergy, asthma, or other atopy-associated diseases.

**Study/Reference**	**Population**
Dong et al. [26] (Wuhan, China)	Case series of 11 patients with COVID-19, 3 of them with a history of allergic disease (1 allergic rhinitis, 1 atopic dermatitis, 1 urticaria)
Bhatragu et al. [25](Seattle, WA, USA)	Report of 3 patients taking oral glucocorticosteroids because of breathing difficulties due to COVID-19 and known asthma who were hospitalized 1 week later with acute respiratory insufficiency
Wang et al. [27] (Wuhan, China)	2 of 69 studied patients had asthma
Zhang et al. [8] (Wuhan, China)	Study of 140 patients of whom 2 had chronic urticaria, 1 had asthma, and 10 had unclear adverse drug reactions
Grasselli et al. [29] (Lombardy, Italy)	Study including 1,591, of whom 205 had a history of: bronchial asthma, anemia, inflammatory bowel disease, chronic respiratory insufficiency, endocrine disorders, chronic pancreatitis, diseases of the connective and supporting tissue, organ transplantation, epilepsy, neurological disease (reported as “other” in the study)
Dreher et al. [28] (Aachen, Germany)	Result: COVID-19 patients with a history of respiratory disease develop ARDS more frequently (58 vs. 42%; 14 vs. 11 patients; of these, 4 vs. 2 patients with asthma; n = 50)

ARDS = acute respiratory distress syndrome.

**Table 3. Table3:** Biologicals approved in Germany, Austria, Luxembourg, and Switzerland for use in allergic diseases.

**Agent**	**Target**	**Indication**	**Self-administration**
Omalizumab Xolair^®^	IgE	IgE-mediated asthma (≥ 6 years) Chronic spontaneous urticaria (≥ 12 years)	Yes (Switzerland: No)
Mepolizumab Nucala^®^	IL-5	Asthma (≥ 6 years; Switzerland: > 12 years) EGPA (only in Switzerland: ≥ 18 years)	Yes
Reslizumab CINQAERO^®^	IL-5	Asthma (≥ 18 years)	No
Benralizumab Fasenra^®^	IL-5 receptor	Asthma (≥ 18 years)	Yes
Dupilumab Dupixent^®^	IL-4 receptor (shared IL-4/IL-13 receptor)	CRSwNP (≥ 18 years) Atopic dermatitis (≥ 12 years) Asthma (≥ 12 years) (Switzerland: approval pending)	Yes

EGPA = eosinophilic granulomatosis with polyangiitis; CRSwNP = chronic rhinosinusitis with nasal polyps.

**Table 4.** Airway infections as an adverse event in phase 3 studies, meta-analyses, and long-term trials (from Vultaggio et al. Allergy 2020 [66]).

Agent	Indication	**Interval/dosage**	**Study (n)**	**Adverse events biological/placebo (n/group)**
Benralizumab (anti-IL-5R)	Severe, uncontrolled asthma	Q4W + placebo, Q4W + Q4W, Q8W + placebo, Q8W + Q8W	Busse et al. (n = 1,576*)	VURTI 15 – 16%/14 – 15%** (1,030/546) URTI 6%/7 – 8% Pnx < 1 – 1%
Dupilumab (anti-IL-4Rα)	Atopic dermatitis	Various (QW, Q2W, Q4W, Q8W, placebo)	Worm et al. (n = 422)	URTI 5.7 – 8.3/7.3 IFZ 0 – 5.7/1.2 HSV1 1.8 – 6/3.7 VURTI 0 – 1.2/3.7
		200 (adolescents)/300 mg Q2W, 300 mg Q4W, placebo	Simpson et al. (n = 250)	URTI 7.2 – 12.2/17.6 HSV 1.2 – 4.8/3.5
		300 mg QW/Q2W, placebo	Simson et al. (n = 1,379)	URTI 3 – 5/2 HSV 0 – 3/1 HSV1 2 – 4/2 HSV2 1/1 VZV 0 – 1/1
		300 mg QW/Q2W, placebo	Blauvelt et al. (n = 740)	URTI 10 – 14/10 IFZ 3 – 4/5 HSV 2 – 3/1 VZV < 1 – 1/2 HSV1 4 – 5/3
		300 mg Q2W (open label)	Faiz et al. (n = 241)	URTI 1.2 HSV < 2.4
		300 mg Q2W	Deleuran et al. (n = 1,491)	VURTI 2.5 IFZ 2.1 HSV1 4.3
	CRSwNP	300 mg Q2W, placebo	Bachert et al. (n = 276)	URTI 5.4 – 6.7/12.7
	Moderate to severe, uncontrolled asthma	200/300 mg Q2W, placebo	Castro et al. (n = 1,897)	VURTI 18.2/19.6 URTI 11.6/13.6 IFZ 5.9/8.0
	Severe, steroid-dependent asthma	300 mg Q2W, placebo	Rabe et al. (n = 210)	VURTI 9/18 IFZ 3/6
Mepolizumab (anti-IL-5)	Severe eosinophilic asthma	75 mg IV Q4W/ 100 mg SC Q4W	Ortega et al. (n = 576)	IFZ 5/3 (191/191), 3/3 (194/191) VURTI 1/< 1, 0/< 1 HSV1 < 1 all HSV2 < 1/0, < 1/0 VZV < 1/0 – 1/0
		100 mg Q4W	Chupp et al. (n = 551)	IFZ 3/1 (273/278) HSV 1 < 1/0 VZV < 1/< 1
	Severe, steroid-dependent eosinophilic asthma	100 mg SC Q4W	Bel et al. (n = 135)	IFZ 4/2 (69/66) VURTI 1/2 VZV 0/2
Reslizumab (anti-IL-5)	Severe eosinophilic asthma	3 mg/kg IV Q4W	Virchow et al. (n = 1,758)	URTI 9/9 (1,028/730) IFZ 3/5
Omalizumab (anti-IgE)	Severe allergic asthma	Q2W/Q4W	Esquivel et al. (n = 327)	Rhinovir 3.3/3.4 (243/84)

*Multi-step design. Total number of all three sub-studies; **patients first received placebo, then active drug. Due to large differences in group sizes percentages are given. Pnx = pneumonia; CRSwNP = chronic rhinosinusitis with nasal polyps; HSV = herpes simplex virus; IFZ = influenza; (V)URTI = (viral) upper respiratory tract infections; VZV = varicella zoster virus.
